# Comparison of the Degree of Chest CT Scan Abnormalities in COVID-19 and Influenza Patients

**DOI:** 10.7759/cureus.75536

**Published:** 2024-12-11

**Authors:** Shiv Goel, Julia Kipp, Adam Kipp, Shelly Jain, Nirmit Goel

**Affiliations:** 1 Public Health, Saint Louis University, St. Louis, USA; 2 Medicine, St. Ignatius College Prep, Chicago, USA; 3 Engineering, Northwestern University, Evanston, USA; 4 Diagnostic Radiology, Shelly Jain MD PC, Oak Brook, USA; 5 Radiology, Michigan State University, East Lansing, USA

**Keywords:** chest imaging, computed tomography, covid-19, emergency department, influenza, x-ray

## Abstract

Introduction

In the emergency department (ED), COVID-19 and influenza are two common viral diseases. They cause similar symptoms in the respiratory system, and most patients' symptoms are relatively mild. We have reported previously that COVID-19 and influenza infections cause similar abnormalities in chest X-ray readings in the ED. Chest X-ray is a convenient, cost-effective, and useful tool, but it is not as sensitive as computed tomography (CT) scans and does not reveal a high level of detail. To assist physicians in obtaining the most advantageous and specific data to guide the diagnosis and treatment of these diseases, this study aimed to compare the degree of abnormalities on chest CT scans between COVID-19 and influenza patients when they were evaluated in the ED.

Methods

From a general diagnostic radiologist's teaching files, 87 chest CT scans of COVID-19 patients and 87 chest CT scans of influenza patients were collected. Based on our initial review, four severity categories of lung abnormalities were established. These four categories were normal, mildly abnormal, moderately abnormal, and severely abnormal. Each CT scan was categorized into one of these four categories after being evaluated by two independent raters. The number of CT scans in each category was then counted for the COVID-19 and influenza groups. The resulting number was also divided by the total number of CT scans in each disease group to obtain the percentage within each category. Finally, the results were compared between the COVID-19 and influenza groups.

Results

In the COVID-19 group, the number and percentage of CT scans in each of the four categories were 10 (11.5%) normal, 44 (50.6%) mildly abnormal, 19 (21.8%) moderately abnormal, and 14 (16.1%) severely abnormal. In the influenza group, there were 13 (14.9%) normal, 48 (55.2%) mildly abnormal, 15 (17.3%) moderately abnormal, and 11 (12.6%) severely abnormal. Chi-square tests revealed no significant difference in these two groups' chest CT abnormalities severity levels.

Conclusion

Our results indicate that most COVID-19 and influenza patients had mild to moderate abnormalities on their chest CT scans at the time of their ED visits, and the overall severity levels of chest CT abnormalities were similar in both groups of patients.

## Introduction

Coronavirus disease 2019 (COVID-19) is primarily a respiratory illness caused by the highly transmissible severe acute respiratory syndrome coronavirus 2 virus (SARS-CoV-2). COVID-19 first surfaced on December 12, 2019, and was declared a pandemic by the World Health Organization (WHO) on March 11, 2020 [1-3]. On May 5, 2023, more than three years since its onset, WHO announced the transition of COVID-19 classification from a public health emergency of international concern (PHEIC) to an established and ongoing health issue and updated its Strategic Preparedness and Response plan accordingly for 2023-2025 [4]. Although the PHEIC has ended, COVID-19 remains a threat to public health. From March 10, 2023, to October 27, 2024, the total number of confirmed cases has risen from 676.80 million to 776.75 million, and the total number of deaths has risen from 6.88 million to 7.07 million worldwide [5,6]. Currently, there are still at least hundreds of thousands of cases and thousands of deaths reported to WHO every month around the world [6]. The SARS-CoV-2 virus that causes COVID-19 is highly mutative and can infect individuals who have had immunity from vaccination or prior exposure. Therefore, better disease prevention and treatment and more efficient long-term COVID-19 control and management are still pressing issues in our societies.

COVID-19 infection can cause various symptoms in patients, ranging from asymptomatic to typical respiratory symptoms. In more severe cases, the infection may result in pneumonia, respiratory failure, or death [7-9]. Similarly, these symptoms can also be observed in patients infected with another common respiratory virus, influenza [2,10-12]. We have reported previously that there is no difference in the degree of chest X-ray abnormalities between COVID-19 and influenza patients [13]. Chest X-ray is a convenient, cost-effective, and useful tool, but it is not as sensitive as computed tomography (CT) scans and does not reveal a high level of detail. Since 2020, some studies have analyzed the use of chest CT scans in assessing COVID-19. However, these studies mostly focused on describing the types of abnormalities, and there has not been a commensurate level of investigation comparing the extent of lung abnormalities for both diseases. Therefore, this study aimed to systematically evaluate, classify, and compare the degree of abnormal findings on the chest CT scans of COVID-19 patients with those of influenza patients. Findings from this study will assist physicians in the ED to determine the extent of lung abnormalities and, therefore, prioritize patients for optimal treatment to attain the best outcomes.

## Materials and methods

The image collection and analysis methods were based on our previous publication [13]. More specifically, from the teaching files collected by a general diagnostic radiologist, a total of 174 chest CT scans from 87 ED patients with a provided history of COVID-19 and 87 ED patients with a provided history of influenza were collected. Both the initial teaching files of the general diagnostic radiologists and the images we obtained for this study were collected randomly based on clinical diagnosis. The randomization was achieved using the built-in RAND function of Excel. The diagnosis of COVID-19 or influenza was based on PCR test results. All patient identification information was removed from the files before the collection. These chest CT scans were from EDs of four community hospitals in the same region of the United States. The CT scans were taken between March 2021 and October 2021 (COVID-19) or between September 2019 and November 2019 (influenza), and the diagnosis of each patient was listed as "COVID-19 positive" or "influenza-positive". We do not have information on the virus strains nor access to patient medical records. Chest CT scans of patients with other types of diagnoses, for instance, "pneumonia", "influenza-like symptoms", and "bacterial pneumonia", were excluded from this study. For the influenza group, we only included patients who had a clear history of "influenza positive" at the time of ED visits.

The collected CT scans were numbered and analyzed on a Windows 10 operating system PC. Two high-resolution monitors were used side by side to maximize the image size and quality. The General Electric Universal Viewer Software was used for the viewing and analysis. An initial review was conducted to achieve a general impression of what the images looked like and the scope and types of lung abnormalities. Next, the methods and criteria of CT scan characterization and categorization were decided. Four severity levels of abnormalities were established, which are normal, mild abnormal, moderate abnormal, and severe abnormal (see details in the Results section) [13].

To categorize the severity levels of lung abnormalities of the CT scans, two independent raters reviewed each scan in detail. The raters did not know each patient's diagnosis before reviewing. Each CT scan was assigned to one of the above-mentioned severity categories. Inter-rater variation was very low. If there was a discrepancy, an experienced radiologist was consulted to help determine the final categorizing decision. After all 174 CT scans were categorized, the number of CT scans in each category in each disease group was counted, and the percentage was calculated compared to the total number of CT scans in that group. Finally, the results were compared between the COVID-19 and the influenza groups.

Statistical analysis

The chi-square test was used to analyze whether the number or percentage of CT scans in the four severity categories was statistically different between the COVID-19 and influenza groups. The chi-square and p-values were calculated using Excel data analysis and the Prism-GraphPad software. P<0.05 was considered significant.

## Results

Evaluation and categorization of chest CT scan findings in COVID-19 patients

As the first step of the image evaluation process, a basic review of the 87 chest CT scans of patients with a provided history of COVID-19 was conducted. The scans were acquired during the pandemic between March 2021 and October 2021. Patient gender distribution was 45% female and 55% male, and patient ages ranged from 20 to 82 years. All CT scans were performed with patients in the supine position (lying on their back). During this initial review of each CT scan, a general familiarity with the imaging appearance of the chest (particularly pulmonary parenchyma) and the range of abnormalities was obtained.

We observed different types of opacities on CT scans, including multifocal, mainly peripheral involvement with ground-glass opacification (GGO), reticulonodular opacification, nodules, alveolar opacification, including consolidation and "crazy paving" (a network of smooth linear patterns superimposed upon an area of ground-glass opacification) [14]. GGO is the most common abnormality [15,16]. As this study focused on parenchymal changes for assessing severity, the presence of pleural effusion (fluid accumulation along the lungs' outer lining) was not included in determining the severity, as effusions are not commonly associated with COVID-19 [17].

**Figure 1:**
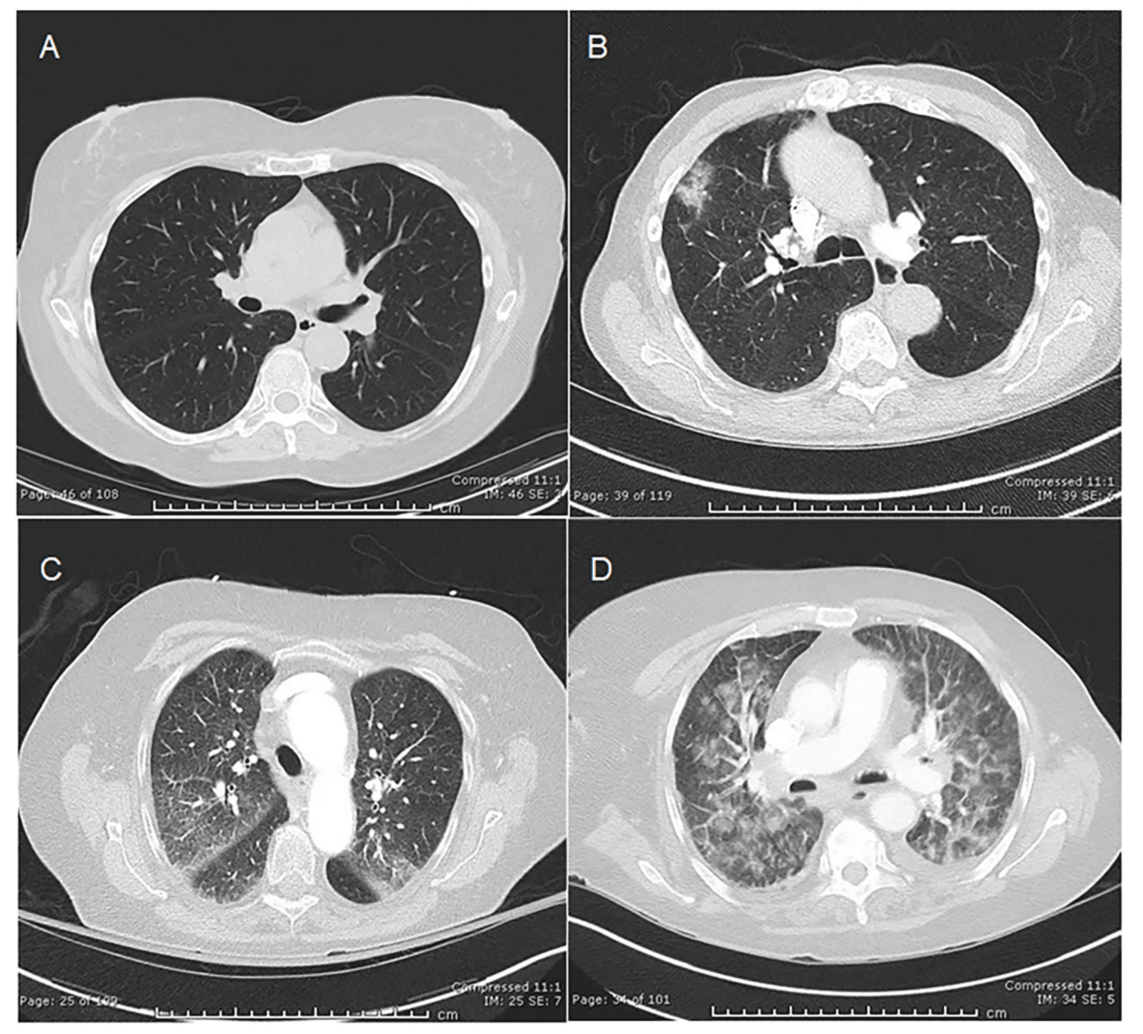
Representative chest CT scans in COVID-19 patients. A) Normal, B) Mildly abnormal, C) Moderately abnormal, and D) Severely abnormal

Based on this evaluation and the approaches used by other investigators [18-20], we classified the 87 chest CT scans of COVID-19 patients into four categories: normal, mildly abnormal, moderately abnormal, and severely abnormal. The following definitions were utilized to guide our assessment. Normal lungs are clear without opacification. Mildly abnormal lungs show opacification affecting less than 25% of one or both lungs, typically in the perihilar regions (adjacent to the heart), with mild unilateral or bilateral involvement. Moderately abnormal lungs demonstrate opacification involving 25-50% of one or both lungs or moderate perihilar opacification. Severely abnormal lungs exhibit extensive opacification exceeding 50% in one or both lungs, prominent perihilar opacification, or multifocal ill-defined opacifications throughout both lungs. Representative CT scan images illustrating these categories are provided in Figure 1.

Evaluation and categorization of chest CT scan findings in influenza patients

A basic review of the 87 chest CT scans of patients with a provided history of influenza was also conducted similarly to the COVID-19 chest CT scans. The scans were acquired between September 2019 and November 2019, before the first case of COVID-19 was identified in the U.S. on January 17, 2020 [21]. Therefore, the influenza cases from these months were deemed to not coincide with COVID-19. Patient gender distribution was 45% female and 55% male, and patient ages ranged from 20 to 82 years. All CT scans were performed with patients in the supine position (lying on their back). During this initial review of each CT scan, a general familiarity with the imaging appearance of the chest (particularly in the pulmonary parenchyma) and the range of abnormalities was obtained.

Similarly to the CT scans of COVID-19 patients, we observed different types of opacities in these influenza CT scans, including GGO, reticulonodular opacification, nodules, alveolar opacification, including consolidation, and "crazy paving". A central or peribronchovascular location appeared more often in influenza scans than in COVID-19 scans. GGO appeared most frequently [2,7,10]. Similarly to the above-mentioned COVID-19 scan analysis, since this study focused on parenchymal changes for assessing severity, pleural effusion (fluid accumulation along the lungs' outer lining) was excluded due to effusions not commonly associated with influenza and appearing to have no strong correlation to clinical outcome [22].

**Figure 2:**
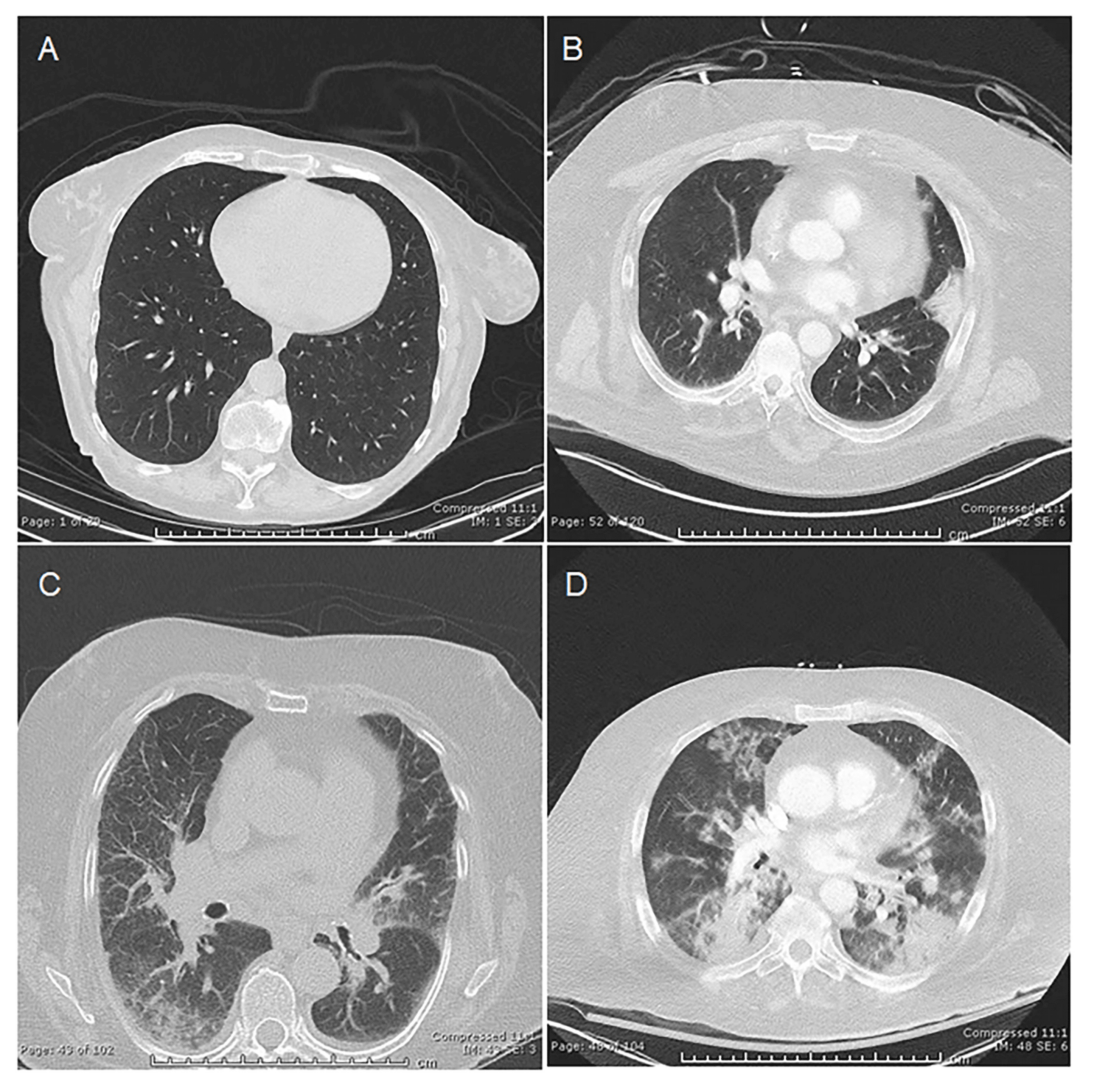
Representative chest CT scans in influenza patients. A) Normal, B) Mildly abnormal, C) Moderately abnormal, and D) Severely abnormal

Based on the initial review of the CT scans and the approaches used by other investigators [9,11,23], we also classified the 87 chest CT scans of influenza patients into four categories: normal, mildly abnormal, moderately abnormal, and severely abnormal. The following definitions were utilized to guide our assessment. Normal lungs are clear without opacification. Mildly abnormal lungs show opacification affecting less than 25% of one or both lungs, typically in the perihilar regions (adjacent to the heart), with mild unilateral or bilateral involvement. Moderately abnormal lungs demonstrate opacification involving 25-50% of one or both lungs or moderate perihilar opacification. Severely abnormal lungs exhibit extensive opacification exceeding 50% in one or both lungs, prominent perihilar opacification, or multifocal ill-defined opacifications throughout both lungs. Representative CT scan images illustrating these categories are provided in Figure 2.

Comparison of degree of abnormalities on chest CT scans between COVID-19 and influenza patients

After the initial evaluation of the CT scans and the determination of classification criteria, each CT exam was closely analyzed and assigned to the appropriate category based on the severity of the findings observed by two independent raters. The number of CT scans in each category was then tallied, and the results were summarized using absolute numbers and percentages. The results showed the distribution of severity categories for COVID-19 patients as follows: Normal in 10 patients (11.5%), mild in 44 (50.6%), moderate in 19 (21.8%), and severe in 14 (16.1%) (Table 1-2).

**Table 1:** The number of CT scans in each severity category in COVID-19 and influenza patients Patients had their CT scans taken during ED visits between March and October 2021 (COVID-19) or between September and November 2019 (influenza) at four community hospitals in the same geographic region in the U.S. The chi-square test was used to examine the association. The chi-square and p-values were calculated using Excel data analysis and the Prism-GraphPad software. χ2: chi-square value; DF: degree of freedom; p < 0.05 was considered statistically significant

Categories	COVID-19 Group (n=87)	Influenza Group (n=87)	Χ^2^ (DF, p-value)
Normal	10	13	2.91 (3, P=0.408)
Mild Abnormal	44	48	
Moderate Abnormal	19	15	
Severely Abnormal	14	11	

Similarly, each influenza patient's CT scan was also analyzed and assigned to the appropriate category based on the severity of the findings rated by two independent raters. The number of CT scans in each category was tallied, and the results were summarized using absolute numbers and percentages. These results showed the distribution of severity categories as follows: normal in 13 patients (14.9%), mild in 48 (55.2%), moderate in 15 (17.3%), and severe in 11 (12.6%) (Table 1-2).

**Table 2:** The percentage of CT scans in each severity category in COVID-19 and influenza patients Patients had their CT scans taken during ED visits between March and October 2021 (COVID-19) or between September and November 2019 (influenza) at four community hospitals in the same geographic region in the U.S. The chi-square test was used to examine the association. The chi-square and p values were calculated using Excel data analysis and the Prism-GraphPad software. χ2: Chi-square value; DF: Degree of freedom; p < 0.05 was considered statistically significant.

Categories	COVID-19 Group (n=87)	Influenza Group (n=87)	Χ^2^ (DF, p-value)
Normal	11.50%	14.90%	3.30 (3, P=0.348)
Mild Abnormal	50.60%	55.20%	
Moderate Abnormal	21.80%	17.30%	
Severely Abnormal	16.10%	12.60%	

After obtaining the categorization for each group, the number and percentage of CT scans in each category between the COVID-19 and the influenza groups were compared. As shown in Table 1, CT scans were normal in 10 and 13 COVID-19 and influenza patients, respectively; mildly abnormal in 44 and 48 patients, respectively; moderately abnormal in 19 and 15 patients, respectively; and severely abnormal in 14 and 11 patients, respectively. The chi-square test showed no significant difference between COVID-19 and influenza groups in the number of CT scans in the four categories. A comparison of the percentage of abnormalities in the four categories confirmed these findings and revealed no significant difference between these two groups (Table 2).

## Discussion

This study compared the degree of abnormalities on chest CT scans from patients with COVID-19 and patients with influenza in the ED setting. The results demonstrated a similar degree of abnormalities on the chest CT scans from both groups, with the majority being mild to moderately abnormal. These results are consistent with the known characteristics of both diseases in that they primarily affect the respiratory system and cause comparable respiratory symptoms. These results are also consistent with reports by others showing that most COVID-19 or influenza patients have mild or moderate symptoms with mild to moderate chest CT scan abnormalities [20,21,23,24].

We have reported previously that there is no significant difference in chest X-ray findings between COVID-19 and influenza patients [13]. Obtaining chest X-rays was a relatively routine practice for patients in the ED with suspected COVID-19, particularly in the initial phase of the pandemic, where some nations relied on abnormal chest X-ray findings as the lone diagnostic evaluation for COVID-19 before the widespread availability of laboratory testing [25]. Throughout the pandemic and after the pandemic, the use of CT scans has accelerated and widely spread, making this current study a necessary extension of our previous work.

Consistent with our previous findings on chest X-rays, results from this study suggest that CT scans should not be used as a technique to conclusively differentiate COVID-19 from influenza. Instead, CT is an excellent supplemental tool to more precisely identify, characterize, and classify the extent of lung abnormalities in both COVID-19 and influenza as compared to chest X-rays due to its superior resolution, resultant demonstration of anatomy, ability to depict additional pulmonary pathologies, and ability to display other myriad structures in the chest and therefore reveal disease which might be lurking within those structures. CT scans can greatly assist physicians in immediately identifying patients with significant lung abnormalities and more severe symptoms, closely monitoring the progression of illness in these at-risk patients, and initiating early treatments to prevent a worse prognosis, such as being admitted to the intensive care unit (ICU) where mortality rates are higher [18,26,27]. Our findings can help decrease potentially non-essential chest CT scans in those with only mild symptoms, leading to lower medical costs, diminished patient radiation exposure, and more effective allocation of limited resources for better diagnosis and treatments. Our findings can also benefit COVID-19 patients by alleviating their concerns and fears, especially in the ED setting, which can be bewildering and frightening for patients.

The strengths of this study include the novel and important questions we asked, the potential clinical applications of the findings, the clear classification criteria we established, and strict data analysis. Although this study followed strict scientific standards and procedures, it had some limitations. First, it was observational and retrospective and relied on interpreting CT scan images, which can be subjective. Second, from a clinical perspective, disparities in medical decisions by physicians in the ED among different hospitals might influence which patients receive CT scans, potentially resulting in an inaccurate representation of patient populations. Third, as SARS-COV-2 is a relatively new virus and our knowledge of it is still advancing, there may be other features or patterns of COVID-19 on CT scans that are not known at this time, and therefore, one would not be expected to recognize them. Fourth, the stage of infection at the time of CT scanning in the ED and the patient's clinical history were not available to this study, which could limit the interpretations of CT scans because underlying conditions, such as chronic lung disease, heart failure, or other pulmonary infections, can appear similarly to the image findings of COVID-19 and influenza. Knowing the stage of infection would also help predict the prognosis and the duration of the disease. Lastly, instead of acute COVID-19, some patients might develop Long COVID, a chronic condition that appears weeks or months after COVID-19 infection [28]. Long COVID-19 could affect patient diagnosis and CT scan results in the ED. For future studies, the limitations mentioned above may potentially be overcome or minimized through advanced planning and coordination, for example, gaining access to patient's medical records, keeping records of virus strain information, acquiring chest CT scans from a consistent physician cohort, adding new features or patterns of COVID-19 on CT scans into severity categories as they are identified by the scientific community, keeping track of patients with long COVID, etc.

This study represents a logical extension of our previous work on chest X-rays as it utilizes a more precise imaging modality to provide significant and meaningful advancement in the field. In the future, we will continue CT scan investigations of COVID-19, influenza, and other respiratory diseases by incorporating the use of artificial intelligence (AI). CT scans contain anatomical information of the lungs beyond that of chest X-rays, and AI can help achieve even more sophisticated imaging analysis by identifying data points not discernible by the human eye [16,29,30]. Therefore, the combined use of CT scans and AI has the potential to identify new imaging features of COVID-19 that are not yet discovered. The combined use of these two technologies also makes it possible to integrate other information, such as medical records and COVID-19 variants, with CT scan findings, providing more comprehensive analysis and suggestions to physicians for quicker and better disease diagnosis and treatment.

## Conclusions

This study provides valuable information to the field by categorizing and comparing the degree of abnormal findings in COVID-19 and influenza patients. The results from this study are consistent with our previous report based on chest X-rays and suggest that CT scans should not be used alone as a tool to distinguish COVID-19 and influenza. Recognizing that most patients demonstrate mild to moderate symptoms and CT scan abnormalities, our findings will not only assist patients in overcoming anxiety but also help ED physicians choose cost-effective treatment plans. In addition, this study provides further validation of the use of chest CT scans in discovering fine pulmonary changes following COVID-19 or influenza infections. Finally, this study helps build a solid foundation for future cutting-edge applications of AI technologies to CT scans in diagnosing and treating COVID-19, influenza, and other respiratory diseases.

## References

[1] Inui S, Gonoi W, Kurokawa R (2021). The role of chest imaging in the diagnosis, management, and monitoring of coronavirus disease 2019 (COVID-19). Insights Imaging.

[2] Yin Z, Kang Z, Yang D, Ding S, Luo H, Xiao E (2020). A comparison of clinical and chest CT findings in patients with influenza A (H1N1) virus infection and coronavirus disease (COVID-19). AJR Am J Roentgenol.

[3] WHO (2020 (2024). Archived: WHO Timeline - COVID-19. https://www.who.int/news/item/27-04-2020-who-timeline---covid-19.

[4] (2024). Statement on the fifteenth meeting of the IHR (2005) Emergency Committee on the COVID-19 pandemic. Published May.

[5] (2024). COVID-19 dashboard. https://gisanddata.maps.arcgis.com/apps/dashboards/bda7594740fd40299423467b48e9ecf6.

[6] WHO. (2024 (2024). About the WHO COVID-19 dashboard. https://data.who.int/dashboards/covid19/about.

[7] Shuai W, Chen X, Shan Y, Li W, Ma W, Lu Q, Li D (2021). Clinical characteristics and CT findings in 148 non-COVID-19 influenza-like illness cases: A retrospective control study. Front Public Health.

[8] Stogiannos N, Fotopoulos D, Woznitza N, Malamateniou C (2020). COVID-19 in the radiology department: What radiographers need to know. Radiography (Lond).

[9] Koo HJ, Lim S, Choe J, Choi SH, Sung H, Do KH (2018). Radiographic and CT features of viral pneumonia. Radiographics.

[10] Samir A, Naguib NN, Elnekeidy A, Baess AI, Shawky A (2021). COVID-19 versus H1N1: Challenges in radiological diagnosis—comparative study on 130 patients using chest HRCT. Egypt J Radiol Nucl Med.

[11] Onigbinde SO, Ojo AS, Fleary L, Hage R (2020). Chest computed tomography findings in COVID-19 and influenza: A narrative review. Biomed Res Int.

[12] Amanullah S, Shapiro M, Dinescu D (2020). Viral pneumonia imaging. Radiology.

[13] Goel S, Kipp A, Goel N, Kipp J (2022). COVID-19 vs. influenza: A chest x-ray comparison. Cureus.

[14] Johkoh T, Itoh H, Müller NL (1999). Crazy-paving appearance at thin-section CT: Spectrum of disease and pathologic findings. Radiology.

[15] Park J, Freer R, Stevens R, Soneji N, Jones N (2021). The accuracy of chest CT in the diagnosis of COVID-19: An umbrella review. Centre for Evidence-Based Medicine.

[16] Gashi A, Kubik-Huch RA, Chatzaraki V (2021). Detection and characterization of COVID-19 findings in chest CT: Feasibility and applicability of an AI-based software tool. Medicine (Baltimore).

[17] Malguria N, Yen LH, Lin T, Hussein A, Fishman EK (2021). Role of chest CT in COVID-19. J Clin Imaging Sci.

[18] Zayed NE, Bessar MA, Lutfy S (2021). CO-RADS versus CT-SS scores in predicting severe COVID-19 patients: Retrospective comparative study. Egypt J Bronchol.

[19] Al-Mosawe AM, Abdulwahid HM, Fayadh NA (2021). Spectrum of CT appearance and CT severity index of COVID-19 pulmonary infection in correlation with age, sex, and PCR test: an Iraqi experience. Egypt J Radiol Nucl Med.

[20] Yang R, Li X, Liu H (2020). Chest CT severity score: An imaging tool for assessing severe COVID-19. Radiol Cardiothorac Imaging.

[21] Hamilton JJ, Turner K, Lichtenstein Cone M (2021). Responding to the pandemic: Challenges with public health surveillance systems and development of a COVID-19 national surveillance case definition to support case-based morbidity surveillance during the early response. J Public Health Manag Pract.

[22] Garrana SH, Som A, Ndakwah GS (2021). Comparison of chest CT findings of COVID-19, influenza, and organizing pneumonia: A multireader study. AJR Am J Roentgenol.

[23] Lin L, Fu G, Chen S, Tao J, Qian A, Yang Y, Wang M (2021). CT manifestations of coronavirus disease (COVID-19) pneumonia and influenza virus pneumonia: A comparative study. AJR Am J Roentgenol.

[24] Saeed GA, Gaba W, Shah A (2021). Correlation between chest CT severity scores and the clinical parameters of adult patients with COVID-19 pneumonia. Radiol Res Pract.

[25] Salehi S, Abedi A, Balakrishnan S, Gholamrezanezhad A (2020). Coronavirus disease 2019 (COVID-19) imaging reporting and data system (COVID-RADS) and common lexicon: A proposal based on the imaging data of 37 studies. Eur Radiol.

[26] Yazdi NA, Ghadery AH, SeyedAlinaghi S (2021). Predictors of the chest CT score in COVID-19 patients: A cross-sectional study. Virol J.

[27] Mruk B, Plucińska D, Walecki J, Półtorak-Szymczak G, Sklinda K (2021). Chest computed tomography (CT) severity scales in COVID-19 disease: A validation study. Med Sci Monit.

[28] CDC. (2024 (2024). Long COVID basics. https://www.cdc.gov/covid/long-term-effects/index.html.

[29] Dou Q, Liu J, Zhang W (2021). Chest CT images for COVID-19: Radiologists and computer-based detection. Front Mol Biosci.

[30] Machnicki S, Patel D, Singh A (2021). The usefulness of chest CT imaging in patients with suspected or diagnosed COVID-19: A review of literature. Chest.

